# Editorial: Exercise and diet: strategies and prescriptions to improve mental and cognitive health

**DOI:** 10.3389/fpsyt.2023.1347233

**Published:** 2024-01-04

**Authors:** Shengyan Sun, Paulo A. S. Armada-da-Silva, Yongcong Shao

**Affiliations:** ^1^School of Physical Education, Huzhou University, Huzhou, China; ^2^Faculdade de Motricidade Humana, Universidade de Lisboa, Lisbon, Portugal; ^3^CIPER, Faculdade de Motricidade Humana, Universidade de Lisboa, Lisbon, Portugal; ^4^School of Psychology, Beijing Sport University, Beijing, China

**Keywords:** exercise, diet, mental health, depression, stress

## Introduction

In recent decades, there has been a notable rise in the prevalence of mental disorders, encompassing an elevated occurrence of depression, anxiety, schizophrenia, and bipolar disorder. As of 2019, one in every eight individuals, totaling 970 million globally, grappled with mental disorders, with anxiety and depressive disorders emerging as the most prevalent (1). The year 2020 witnessed a substantial escalation in the number of individuals living with anxiety and depressive disorders, attributable to the COVID-19 pandemic. Preliminary estimates reveal a 26 and 28% upswing, respectively, for anxiety and major depressive disorders within a single year (2). Consequently, mental disorders stand among the foremost contributors to the global non-fatal disease burden, presenting a substantial challenge to health systems (3, 4). Internationally, mental health is acknowledged as a key focus in health policies and has been incorporated into the Sustainable Development Goals (4, 5).

The frontline approaches to treat mental disorders are pharmacological treatments and psychotherapy. Nonetheless, for numerous individuals with mental disorders, psychotropic medication does not yield clinically significant long-term improvements, and notable side effects such as substantial weight gain, glucose intolerance, sexual dysfunction, and elevated blood pressure levels, pose considerable challenges (6, 7). These side effects frequently lead to medication discontinuation, causing distress and negatively impacting patients' lives (8).

Consequently, alternative approaches for preventing and treating mental disorders, deliverable alongside or independently of conventional mental health care strategies, are imperative to mitigate the escalating global burden of these conditions. From social and economic perspectives, opting for non-pharmacological interventions (e.g., physical activity and dietary modifications) proves a safer and more cost-effective strategy for enhancing the mental health. A growing body of original studies attests to the capacity of exercise and diet interventions to augment mental health. This review explores areas where exercise and diets are posited to hold potential clinical utility in enhancing mental health, accompanied by a brief discussion of the available evidence.

## Effects of exercise and physical activity on mental health

Several epidemiological studies have indicated a correlation between insufficient physical activity or prolonged periods of sedentary behavior and an increased risk of compromised mental health (9, 10) (Wang et al.). In a recent study involving 1.2 million US adults, where participants were meticulously matched based on various background and demographic factors, those engaging in regular exercise exhibited superior mental health functioning compared to their non-exercising counterparts (9). Similarly, the meta-analyses of 49 prospective studies involving over 260,000 participants revealed a significant link between higher physical activity levels and a reduced risk of depression (OR = 0.83, 95% CI = 0.79–0.88), remaining consistent across age groups, genders, and geographic regions (10). Adjustments for confounding factors, such as weight, smoking, and health conditions, maintained this association (10). Another meta-analysis of 11 studies, with more than 69,000 participants, demonstrated that increased physical activity significantly lowered the incidence of anxiety (OR = 0.74, 95% CI = 0.62–0.88), showing protective effects against specific anxiety disorders like agoraphobia (OR = 0.43, 95% CI = 0.19–0.99) and post-traumatic stress disorder (OR = 0.58, 95% CI = 0.39–0.86) (11). Compared to the high fit counterparts, individuals with low or moderate fitness levels face a 47 and 23% greater risk of experiencing mental health disorders (12). Moreover, a discernible dose-response correlation is evident between increased physical activity and enhanced mental health functioning. Even at a minimal threshold of 20 min/week of physical activity, mental health benefits were evident; however, a dose-response pattern revealed a more pronounced risk reduction with higher volume and/or intensity of activity (13). Taken together, robust epidemiological evidence underscores the protective role of habitual physical activity against the onset of mental health conditions.

Findings from randomized exercise trials, though generally positive, exhibit more variability compared to observational studies (14, 15) (Zhang et al.). A wealth of evidence indicates that exercise, especially aerobic training, alleviates symptoms of depression, anxiety, and stress, yielding treatment effects comparable to conventional psychological or pharmacological approaches (15, 16). A brief 20–40 min of aerobic exercise is reported to enhance mood and alleviate anxiety for several hours, with individuals experiencing acute anxiety showing more positive responses compared to those with chronic anxiety (16, 17). Aerobic exercise trials typically indicate substantial improvements, ranging from moderate to large effect size (ES), when compared to either no intervention (ES = 1.24) or usual care (ES = 0.68) (14). However, these benefits often diminish over time and are generally not statistically significant during follow-up (ES = 0.22) (14), with therapeutic effects only persist among individuals who maintain exercise after the conclusion of active treatment (15). In contrast, engaging in exercise to an extreme extent may lead to mood and behavioral disorders and a decline in physical health (16).

Most studies examining the impact of exercise on mental health predominantly focus on cardiorespiratory conditioning through aerobic exercise that involve sustained engagement of large muscle groups. There is a scarcity of research on other exercise training forms that emphasize muscular strength, flexibility, agility, balance, and coordination, such as resistance training (18), yoga, swimming (19), Qigong, Tai Chi (20), etc. However, these non-aerobic exercises have also demonstrated heightened benefits for mood outcomes. For instance, resistance training paradigms were found to improve depressive symptoms with a moderate-sized mean effect (ES = 0.66, 95% CI = 0.48–0.83) (18). Engagement in yoga and swimming activities significantly improved feelings of anger, confusion, tension, and depression compared to individuals who were in the control group (19). Thus, it seems that mood enhancement is not exclusive to aerobic exercise forms. In a randomized controlled study involving 91 inpatients with major depression, dysthymic disorder or depressive disorder, 8 weeks of both aerobic and non-aerobic exercise regimes resulted in reduced depression scores with no significant group differences (21). Similarly, a study involving 79 participants with anxiety disorders, the impact of aerobic activities (brisk walks or jogging) was compared with non-aerobic activities (muscular strength, flexibility, and relaxation), revealing similar improvements in anxiety scores for both groups (22).

In summary, exercise and regular physical activity are considered to potentially exert a significant impact on mental health, comparable to traditional psychotherapeutic and pharmacological approaches. However, there is a lack of consensus on the ideal amount and type of activity to attain these benefits. Moreover, the effects of exercise training vary across patient populations and training modalities, and the long-term mental health benefits seem to rely on sustained physical activity engagement. Consequently, additional research is imperative to investigate the impact of diverse exercise modalities, frequencies, durations, and intensities on mental health. Understanding the underlying causal mechanisms of treatment response across various patient populations is also essential. These investigations play a pivotal role in establishing the ultimate effectiveness of exercise and physical activities as supplementary treatment.

## Effects of diet and nutrition on mental health

The brain's composition, structure, and function rely on the presence of essential nutrients, such as lipids, amino acids, vitamins, and minerals. Consequently, it is logical to infer that the quality and quantity of food intake could influence brain function. This establishes diet as a modifiable factor that can be addressed to enhance mental health, mood, and cognitive performance. As a matter of fact, “nutritional psychiatry” is a rapidly emerging field that explores the intersection of nutrition and mental health. While establishing a definitive causal relationship between specific diets or dietary components and their impact on mental health—whether in causing, preventing, or treating diseases—is challenging, there is an increasing body of epidemiological studies indicating a clear association between dietary patterns and mental wellbeing. Unhealthy dietary patterns can have adverse effects on mental health, as evidenced by an epidemiological study of 70,190 U.S. adults indicating that a higher pro-inflammatory diet is associated with an increased risk of depression (Luo et al.). In contrast, a healthy diet, characterized by a diet high in whole grains, healthy fats (found in fish, olive oil, nuts, etc.), vegetables, fruits and fish, appears to have a protective effect against common mental disorders (23–25). For example, a randomized controlled trial has demonstrated significant improvements in moderate-to-severe depression following a 12-week modified Mediterranean diet intervention, with the dietary group showing a notably greater improvement and achieving remission in 32.3% of participants (26). Several systematic reviews and meta-analyses also offer compelling evidence that adopting a healthy diet, in particular a Mediterranean diet, can provide protection against mental disorders (27–29).

In addition to cross-sectional and intervention studies on the whole dietary, there have been notable advancements in elucidating the impact of specific nutritional compounds, or nutraceuticals, on mental wellbeing. For instance, the supplementation of Vitamin B6 and Vitamin B12 has been documented to postpone the onset and ameliorate the prognosis of depression (30). Moreover, elevated serum Vitamin D levels have shown a correlation with improved attention and working memory performance in older adults (31). There is also an indication that Vitamin D supplementation might have an impact on depression (32), and attention deficit hyperactivity disorder (33). Additionally, nutraceuticals like docosahexaenoic acid (DHA) and omega-3 polyunsaturated fatty acid serve as crucial structural components in brain membrane phospholipids, ensuring neuronal membrane integrity, fluidity, and functioning (34). These studies offer robust evidence for the efficacy of various nutraceuticals as agents for mood modification and cognitive enhancement across clinical and healthy populations.

The intricate mechanisms through which nutrition impacts the brain are likely to be manifold and complex (a comprehensive review of the underlying mechanisms exceeds the scope of this editorial). Previous mechanistic studies have identified potential cognitive targets for nutraceuticals, encompassing systemic and central vascular function, metabolism, hippocampal neurogenesis, central activation, inflammation, enhanced neural efficiency, and angiogenesis. However, recent research in nutritional psychiatry has explored the modulation of gut microbiota (via probiotics and prebiotics) as an innovative therapeutic approach for neuropsychiatric conditions.

## Conclusions

Considerable progress has been made in understanding the impact of exercise and diet on brain function, as well as the origins and management of related disorders. This study summaries evidence supporting the notion that exercise and a balanced diet can alleviate symptoms related to anxiety, depression, and stress. It is evident that regular exercise and a healthy diet contribute to improved mental wellbeing, providing a practical preventive or adjunctive treatment avenue for improving mental health outcomes. However, much of the evidence is correlational, and there is a deficiency in well-controlled intervention studies with adequate duration and specificity to demonstrate the beneficial effects of exercise and diet on mental health. Moreover, there exists a gap in understanding the causal pathways through which these effects occur. Novel breakthroughs in elucidating the bidirectional relationships between exercise and brain functioning, or nutrition and brain functioning are urgently needed to shape public health policies on exercise and diet. Given the widespread prevalence of mental disorders and the acknowledgment of exercise and diet as modifiable risk factors, even marginal enhancements in physical activities and nutritional contexts have the potential to yield substantial improvements in mental health and overall wellbeing at a population level.

## Author contributions

SS: Conceptualization, Writing—original draft, Writing—review & editing. PA: Writing—review & editing. YS: Writing—review & editing.
